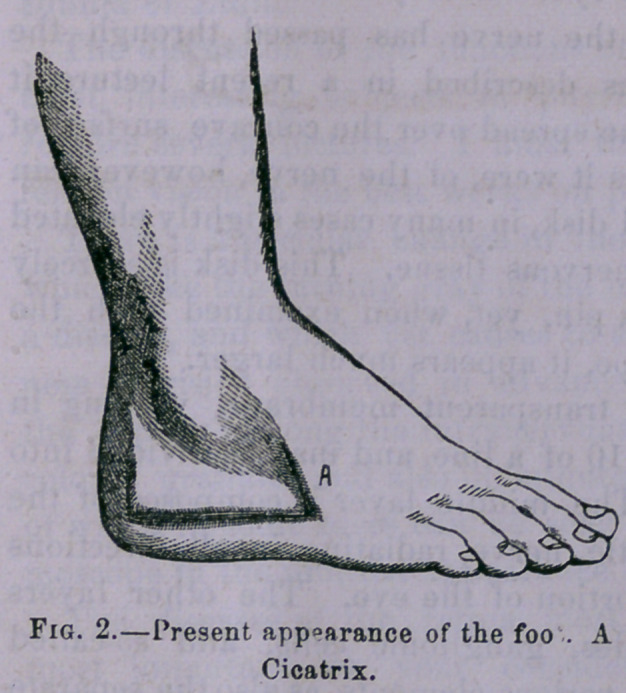# Excision of the Os Calcis and Part of the Astragalus for Caries

**Published:** 1865-09

**Authors:** A. J. Baxter

**Affiliations:** Chicago, Ill.


CHICAGO
MEDICAL JOURNAL.

VOL. XXII.] SEPTEMBER, 1865. [No. 9.

### ORIGINAL COMMUNICATIONS.

### EXCISION OF THE OS CALCIS AND PART OF THE ASTRAGALUS FOR CARIES.

### RECOVERY WITH A PERFECTLY SOUND AND USEFUL FOOT.

By A. J. BAXTER, M. D., of Chicago, Ill.

The very important part which the foot performs in the human economy; its exposed position, and consequent liability to injury, have obtained for it a large share of surgical attention.

The practicability of conservative surgery of the foot appears to have been first demonstrated by Garengeot, and subsequently dilated upon by Heister—but the credit of the first operation was reserved for Du Viviers, in 1783.

Since which, so numerous and ingenious have been the attempts to save as much as possible of this important member, that nearly every bone, from the metatarso-phalangeal articulation to the ankle-joint, has its peculiar operation and champion. Yet in spite of all that has been said in favor of this or that operation, they are not all "surgical fixtures" by any means—as our medical journals and the hotly contested debates of medical societies will bear witness. When doctors disagree, who is to decide? If I may be allowed to answer this question, I would say "Statistics." As the real merits of surgical operations are only obtained by comparing the result of a large number of instances in which they have been performed, it is obviously the duty of every surgeon to place upon record a clear and truthful account of all cases that fall within his observation, that each one may contribute his mite to the mass of experience from which alone correct and final results can be deduced.

Case.—In the early part of January, I was requested by Dr. Payne to see Wm. C———, aged 7 years, who he said had caries of the bones of the right foot, and he feared the ankle-joint was involved also.

At my request, the Doctor has furnished me with the following history of the case:

"When I saw Wm. C——— first, (November 13th, 1864), he had been confined to his bed eight days with what was supposed to be acute rheumatism—but in reality was erysipelas. The scrofulous diathesis was well marked—his father having died of consumption. He was very much emaciated —tongue dry and coated with a heavy brown fur throughout— pulse 140, small and weak—surface hot and dry, with considerable tympanitis, subsultus tendinum, &c., all together presenting rather an unfavorable aspect.

The erysipelas developed itself simultaneously on both superior and inferior extremities—though more marked on the right side. On the right inferior extremity it commenced at the tip of the great toe, and extended four inches above the knee, completely surrounding the limb, with much swelling up to the inguinal region, including the inguinal glands, which were very tender on pressure. On the left extremity it commenced upon the dorsum of the foot, and extended three or four inches above the ankle-joint. The superior extremities were involved about a like covering about five inches over and around each elbow-joint.

Under the liberal use of tincture of iron, quinine, milk-punch and beef-tea he gradually improved, and by the first of December he was able to sit up—the erysipelas had entirely disappeared, but there remained considerable swelling about the right ankle.

December 2d.—Very much improved generally—though the foot and ankle remain somewhat swollen and tender. Ordered all treatment suspended save the iron and poultice to foot, and discontinued my visits.

December 17th.—Was called again, and found that two abscesses had formed. One on the right side external to the nipple—the other below the internal malleoulus of the right foot; opened them, the former discharged a large quantity of pus—the latter very little. After a careful examination of the foot with a probe, came to the conclusion that the abscess was confined to the cellular tissue—as no opening could be found to the bone, ordered dressing of bread and milk poultice to foot, and Syr. Ferri Iod. gtt ter die. At the end of ten days the abscess in the side had entirely healed, the one in the foot appeared nearly so. Again discontinued my visits.

February 25th.—The mother called upon me and exhibited four small pieces of bone which she said had escaped from openings in the foot. Requested Dr. Baxter to see the case with me."

CONDITION AT TIME OF OPERATION.

General health good—says he would be "perfectly well were it not for the foot." The foot and ankle are considerably swollen and club shaped—skin tense glazed and semielastic. There are three sinuses [one in front of the external malleolus—one between it and the heel--the other below the internal malleolus,] from which there is a good deal of sanio-purulent discharge. A probe introduced through any of the openings led to the astragalus and os calcis, which were softened and carious. Owing to the swelling, it was a matter of uncertainty how far either of the bones were involved—but it was thought that the disease was confined principally to the os calcis and the under surface of the Astragalus, which was substantially correct, only both bones were much more carious than was supposed. After informing the mother of the nature of the disease—and that a small incision in the foot would be necessary—she readily consented.

The patient being chloroformed, by Dr. Payne, the operation was commenced by making an incision from the superior external fistulous opening (*See Fig.* 1.)

FIG. 1.—Appearance of the Foot before the Operation. A lines of incision.

downwards and forwards to midway between the calcaneo—cuboid articulation and the base of the metatarsal bone of the little toe—thence backwards to within half an inch of the tuberosity of the os calcis and finally upwards along the anterior border of the tendo achillis to the extent of an inch and a quarter.

After raising this flap or curtain as it were and looking in, such an amount of disease was discovered—especially in the astragalus—that it was thought it would be necessary to convert the operation into one of amputation of the leg. But to determine this matter it was decided to turn out the diseased bones, and see how things would look. Accordingly a small chisel was entered at the calcaneo-cuboid articulation, and the os calcis being very friable was literally "scooped" out. This brought the under surface of the astragalus into full view--and after gouging out the interior of the bone it was found that the entire disease could be removed without opening the articulation—though the crust left was so thin that it caused me great fear for the result of the operation. The tourniquet being loosed, no hemorrhage ensued, the wound was stuffed with lint, a compress and bandage applied, the patient removed to bed and half a grain of opium given. On examining the os calcis, some parts of it seemed to have undergone what Mr. Gant describes as "fatty disintegration," with free oil globules floating about. From this time forward there never was the slighest pain in the foot, or difficulty of any kind.

About two weeks after the operation, his mother took him into the country to visit some relations where he remained about two months. On his return to the city, the wound had entirely healed, and as there was no tenderness, he was running about the str'ts with the aid of a cane. He has frequently been forbidden using the foot so extensively—but as its use is unattended with pain, the injunction goes unheeded. The only sign of the operation having been performed, is a slightly depressed cicatrix [*see Fig.* 2 *A*] on the outside of the foot, with a slight inclination of the foot outwards—the result of his persevering use of the

FIG. 2.—Present appearance of the foot. A Cicatrix.

foot at too early a period. The shortening or flattening of the heel is so trivial that I do not think it will ultimately exceed a quarter of an inch.

## Figures and Tables

**Fig. 1. f1:**
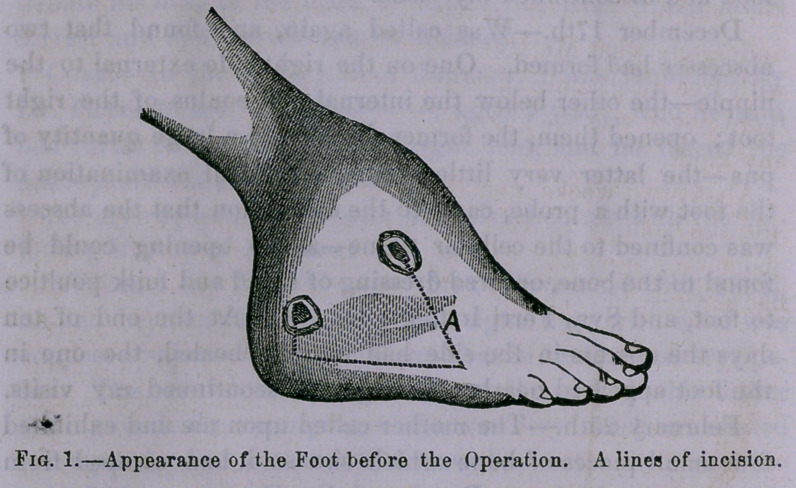


**Fig. 2. f2:**